# Supplementary material for: Placenta Accreta Spectrum Disorder Complicated with Endometriosis: Systematic Review and Meta-Analysis
Source: Biomedicines. 2022 Feb 6;10(2):390. doi: 10.3390/biomedicines10020390 (PMC8962380; doi:10.3390/biomedicines10020390)
Supplement: Supplementary file 1 [file biomedicines-10-00390-s001.zip › Metadata table_123121.pdf]

Supplemental File S1. The search strategy.

PubMed

#1 “In Vitro Fertilization” [TIAB]

#2 fertilization in vitro [MeSH]

#3 Assisted Reproductive Techniques [MeSH]

#4 Embryo Transfer [MeSH]

#5 Intracytoplasmic Sperm Injection [MeSH]

#6 Cryopreserved [TIAB]

#7 "Oocyte donation" [TIAB]

#8 "fresh cycle" [TIAB]

#9 "frozen cycle" [TIAB]

#10 #1 OR #2 OR #3 OR #4 OR #5 OR #6 OR #7 OR #8 OR #9

#11 Endometriosis [MeSH]

#12 endometriosis [TIAB] OR endometrioma [TIAB] OR “chocolate cyst” [TIAB]

#13 adhesion [TIAB] OR “ectopic endometri*” [TIAB]

#14 peritoneal [TIAB] OR abdominal [TIAB] OR pelvic [TIAB] OR Douglas [TIAB] OR “uterine wall” [TIAB] OR “anterior wall” [TIAB] OR “posterior wall” [TIAB] OR abdominal [TIAB] OR ovary [TIAB] OR extrauterine [TIAB] OR extraovarian [TIAB] OR cul-de-sac [TIAB] OR adnexal [TIAB] 635,500

#15 #13 AND #14

#16 #11 OR #12 OR #15

#17 Placenta previa [MeSH]

#18 “placenta previa" [TIAB] OR "low lying placenta" [TIAB]

#19 "abnormal placenta*" [TIAB]

#20 Placenta accreta [MeSH]

#21 “Morbidly adherent placenta" [TIAB] OR "Morbid adherent placenta" [TIAB]

#22 “Placenta accreta spectrum " [TIAB]

#23 “Placenta accreta” [TIAB] OR "Placenta increta" [TIAB] OR "placenta percreta" [TIAB]

#24 "adherence of the placenta" [TIAB] OR "adherent placenta" [TIAB]

#25 #17 OR #18 OR #19 OR #20 OR #21 OR #22 OR #23 OR #24

#26 #16 AND #25

Cochrane Library

#1 “In Vitro Fertilization”:ab,ti,kw

#2 MeSH descriptor: [fertilization in vitro] explode all trees

#3 MeSH descriptor: [Assisted Reproductive Techniques] explode all trees

#4 MeSH descriptor: [Embryo Transfer] explode all trees

#5 MeSH descriptor: [Intracytoplasmic Sperm Injection] explode all trees

#6 Cryopreserved:ab,ti,kw
#7 "Oocyte donation":ab,ti,kw
#8 "fresh cycle":ab,ti,kw
#9 "frozen cycle":ab,ti,kw
#10 #1 OR #2 OR #3 OR #4 OR #5 OR #6 OR #7 OR #8 OR #9
#11 MeSH descriptor: [Endometriosis] explode all trees
#12 endometriosis:ab,ti,kw OR “endometrioma:ab,ti,kw OR “chocolate cyst”:ab,ti,kw
#13 #11 OR #12
#14 adhesion:ab,ti,kw OR ectopic endometrium:ab,ti,kw
#15 peritoneal:ab,ti,kw OR abdominal:ab,ti,kw OR pelvic:ab,ti,kw OR Douglas:ab,ti,kw OR “uterine wall”:ab,ti,kw OR “anterior wall”:ab,ti,kw OR “posterior wall”:ab,ti,kw OR abdominal:ab,ti,kw OR ovary:ab,ti,kw OR extrauterine:ab,ti,kw OR extraovarian:ab,ti,kw OR cul-de-sac:ab,ti,kw OR adnexal:ab,ti,kw
#16 #14 AND #15
#17 #13 OR #16
#18 MeSH descriptor: [Placenta previa] explode all trees
#19 “Placenta previa":ab,ti,kw OR "low lying placenta":ab,ti,kw
#20 "abnormal placenta":ab,ti,kw
#21 MeSH descriptor: [Placenta accreta] explode all trees
#22 “Morbidly adherent placenta":ab,ti,kw OR "Morbid adherent placenta":ab,ti,kw
#23 “Placenta accreta spectrum":ab,ti,kw
#24 "Placenta accreta":ab,ti,kw OR "Placenta increta":ab,ti,kw OR "Placenta percreta":ab,ti,kw
#25 "adherence of placenta":ab,ti,kw OR "adherence of the placenta":ab,ti,kw OR "adherent placenta":ab,ti,kw
#26 #18 OR #19 OR #20 OR #21 OR #22 OR #23 OR #24 OR #25
#27 #17 AND #26

Scopus
#1 TITLE-ABS-KEY ("In Vitro Fertilization")
#2 TITLE-ABS-KEY ("fertilization in vitro")
#3 TITLE-ABS-KEY ("Assisted Reproductive Techniques" OR "Assisted Reproductive Technology")
#4 TITLE-ABS-KEY ("Embryo Transfer")
#5 TITLE-ABS-KEY ("Intracytoplasmic Sperm Injection")
#6 TITLE-ABS-KEY ("Cryopreserved")
#7 TITLE-ABS-KEY ("oocyte donation")
#8 TITLE-ABS-KEY ("fresh cycle")
#9 TITLE-ABS-KEY ("frozen cycle")

#10 #1 OR #2 OR #3 OR #4 OR #5 OR #6 OR #7 OR #8 OR #9

#11 TITLE-ABS-KEY ("endometriosis" OR "endometrioma" OR “chocolate cyst”)

#12 TITLE-ABS-KEY ("adhesion" OR “ectopic endometrium”)

#13 TITLE-ABS-KEY (peritoneal OR abdominal OR pelvic OR Douglas OR “uterine wall” OR “anterior wall” OR “posterior wall” OR abdominal OR ovary OR extrauterine OR extraovarian OR cul-de-sac OR adnexal)

#14 #12 AND #13

#15 #11 OR #14

#16 TITLE-ABS-KEY (“placenta previa” OR “low lying placenta”)

#17 TITLE-ABS-KEY (“abnormal placentation”)

#18 TITLE-ABS-KEY ("Morbidly adherent placenta" OR "Morbid adherent placenta")

#19 TITLE-ABS-KEY ("Placenta Accreta Spectrum")

#20 TITLE-ABS-KEY (“Placenta accreta” OR "Placenta increta" OR "Placenta percreta")

#21 TITLE-ABS-KEY ("adherence of placenta" OR "adherence of the placenta" OR "adherent placenta")

#22 #16 OR #17 OR #18 OR #19 OR #20 OR #21

#23 #15 AND #22

Supplemental Table S1. Meta data of included studies.

Author	Location	Year	Period	Type	Match	Exp	Control	Total	Total No. End	Exp No. End	Control No. End	No. Exp	No. Control	End PAS	ART PP	ART PAS
Wang Y[1]	CHN	2021	2013-2018	Nationwide	--	ART	No ART	16879728	--	--	--	269738	16609990	--	Yes	Yes
Zargar M[2]	IRN	2021	--	Retro	--	Frozen	Fresh	669	--	--	--	356	313	--	Yes	--
Hu KL[3]	CHN	2021	2013-2019	Retro	--	HRC	NC	7021	--	--	--	2561	3790	--	Yes	--
Asserhøj LL[4]	DNK	2021	2006-2014	Retro	--	HRC	NC	1136	33	16	17	357	779	--	Yes	--
Wang J[5]	CHN	2021	2009-2018	Retro	Yes	ART<br>ART_end	No ART<br>ART_non-end	8773	74	74	--	1241	6832[d]	Yes	Yes	Yes
Epelboin S[6]	FRN	2021	2013-2018	Nationwide	--	ART_end<br>SC_end	SC_end<br>SC_non-end	4121767	38035	6934<br>31101	31101<br>0	6934<br>31101	31101<br>4083732	--	Yes	--
Cochrane E[7]	CHN	2020	2015-2018	Retro	Yes	ART	SC	360	--	--	--	120	240[d]	--	Yes	Yes
Modest AM[8]	USA	2020	2013-2018	Retro	--	ART	No ART	28344	--	--	--	1418	26926	--	--	Yes
Salmanian B[9]	USA	2020	2012-2019	Retro	--	ART	No ART	37461	--	--	--	571	36890	--	Yes	Yes
Shinohara S[10]	JPN	2020	2012-2017	Retro	--	ART<br>Frozen	SC<br>Fresh	2583	--	--	--	442<br>330	1764<br>112	--	Yes	--
Farella M[11]	ITA	2020	2009-2016	Retro	--	ART_end	SC_end	535	535	199	336	199	336	--	Yes	--
Petersen SH[12]	NOR	2020	1994-2015	Nationwide	--	ART	SC	6830578	--	--	--	146998	6683580	--	Yes	--
Spangmose AL[13]	NOR	2020	1997-2015<br>Overlapped[12]	Nationwide	--	ART[†]	SC	2864880	--	244	Unk	4601	2808323	--	Yes	--
Tanaka H[14]	JPN	2020	2015-2017	Retro	--	ART	No ART	6952	--	--	--	811	6141	--	Yes	Yes
Kyozuka H[15]	JPN	2019	2011-2014	Nationwide	--	ART	No ART	90554	--	--	--	2658	87896	--	--	Yes
Miura M[16]	JPN	2019	2010-2017	Retro	--	ART	No ART	2769	80	23	57	366	2403	--	Yes	--
Santi E[17]	ITA	2019	2014-2018	Retro	--	ART	No ART	11769	--	--	--	281	11488	--	Yes	--
Sakai Y[18]	JPN	2019	2015-2017	Retro	--	Frozen	Fresh[c]	87	--	--	--	60	27	--	Yes	Yes
Saito K[19]	JPN	2019	2014	Nationwide	--	HRC	NC	34980	--	--	--	24225	10755	--	Yes	Yes
Szymusik I[20]	POL	2019	2004-2014	Retro	Yes	ART	SC	634	--	33	Unk	336	308[d]	--	Yes	--
Yang P[21]	CHN	2019	2015-2018	Retro	Yes	ART	No ART	4989	--	--	--	1663	3326[d]	--	Yes	--
Nagata C[22]	JPN	2019	2011-2014<br>overlapped[15]	Nationwide	--	ART	SC	97592	--	--	--	3147	90506	--	Yes	#
Rahu K[23]	EST	2019	2005-2014	Nationwide	--	ART	SC	35333	--	--	--	1778	33555	--	Yes	--
Shmueli A[24]	ISR	2019	2007-2014	Retro	--	End	Non-end	61535	135	135	61400	135	61400	Yes	--	--
Nirgianakis K[25]	CHE	2018	2004-2015	Retro	Yes	End	Non-end	248	62	62	186	62	186[d]	Yes	--	--
Sabban H[26]	CAN	2017	2008-2011	Nationwide	--	ART	No ART	3315764	--	--	--	5773	3309991	--	Yes	--
Li H[27]	CHN	2017	2011-2013	Retro	Yes	ART_end	No ART_end	375	75	24	74	24	74[d]	--	Yes	--
Berlac JF[28]	DNK	2017	1977-2014	Nationwide	--	End	Non-end	1091251[b]	19331[b]	19331	0	19331	1071920	Yes	--	--
Qin J[29]	CHN	2017	2013-2016	Retro	--	ART	SC	7023	--	--	--	1260	2480	--	Yes	--
Fujii T[30]	JPN	2016	2000-2014	Retro	--	ART_end	ART_non-end	604	92	92	0	92	512	--	Yes	--
Zhu L[31]	CHN	2016	2006-2014	Retro	Yes	ART	SC	7923	--	--	--	2641	5282[d]	--	Yes	Yes
Benaglia L[32]	ITA	2016	2008-2014	Retro	Yes	ART_end	ART_non-end	478	--	--	--	239	239[d]	--	Yes	--
Wennberg AL[33]	DNK	2016	1982-2007<br>Overlapped[12]	Nationwide	--	ART	SC	273536	--	--	--	35571	237965	--	Yes	--
Jacques M[34]	FRN	2016	2009-2014	Retro	Yes	ART_end	ART_non-end	2315	113	113	0	113	113[d]	--	Yes	--
Ginström E[35]	SWE	2016	2002-2013	Nationwide	--	ART<br>Frozen	SC<br>Fresh	1226960<br>30566	--	--	--	30566<br>7795	1196394<br>22771	--	Yes	--
Valenzuela B[36]	ESP	2016	2004-2010	Retro	--	ART	No ART	948	--	--	--	223	725	--	Yes	--
Takeshima K[37]	JPN	2016	2007-2012	Nationwide	--	Frozen	Fresh	141,228	--	--	--	90783	50445	--	Yes	Yes
Kaser DJ[38]	USA	2015	2005-2011	Retro	Yes	Frozen	Fresh	199	--	--	--	42	157	--	--	Yes
Rombauts L[39]	AUS	2014	2006-2012	Retro	--	Frozen	Fresh	4537	316	99	217	1586	2951	--	Yes	--
Korosec S[40]	SVN	2014	2004-2011	Retro	--	Frozen	Fresh	1126	229	42	187	211	915	--	Yes	Yes
Yang X[41]	CHN	2014	2011	Retro	--	ART	SC	112403	--	--	--	1139	111264	--	Yes	--
Ishihara O[42]	JPN	2014	2008-2010	Nationwide	--	Frozen	Fresh	48158	--	--	--	31249	16909	--	Yes	Yes

			overlapped[37]													
Räisänen S[43]	FIN	2013	2006-2010	Nationwide	--	ART	No ART	291004	--	--	--	5647	285357	--	Yes	--
Liu SY[44]	CHN	2013	2006-2011	Retro	--	Frozen[a]	Fresh[a]	16492	--	--	--	1531	1914	--	Yes	--
Takemura Y[45]	JPN	2013	--	Retro	--	ART_end	ART_non-end	318	53	53	0	53	265	--	Yes	--
Sazonova A[46]	SWE	2012	2002-2006 Overlapped[35]	Nationwide	--	Frozen	Fresh	11292	--	--	--	2348	8944	--	Yes	--
Hayashi M[47]	JPN	2012	2001-2005	Nationwide	Yes	ART	SC	25069	--	--	--	4570	4264	--	Yes	Yes
Benaglia L[48]	ESP	2012	2005-2009	Retro	Yes	ART_end	ART_non-end	234	78	78	0	78	156	--	Yes	--
Esh-Broder E[49]	ISR	2011	2004-2009	Retro	--	ART	SC	25193	--	--	--	752	24441	--	--	Yes
						ART	No ART			--	--	6730	24619			
Healy DL[50]	AUS	2010	1991-2004	Nationwide	--	Frozen	Fresh	34295	--	--	--	2503	4227	--	Yes	--
						ART_end	ART_non-end			1265	0	1265	5465			
Verlaenen H[51]	BEL	1995	1988-1994	Retro	Yes	ART	No ART	280	--	--	--	140	140	--	Yes	--
Hill GA[52]	USA	1990	1982-1988	Retro	Yes	ART	No ART	182	--	--	--	61	121	--	Yes	--

† blastocyst transfer; # the data about PAS was not used due to the overlapping data[22]; [a]: day 3 embryo [b]: No. of infants [c]: ovulation cycle [d]: patient background matched control cases. Some values listed above might be slightly different from the original values, as estimated by the authors. Abbreviations: Exp, Experimental; End, endometriosis; PAS, placenta accreta spectrum; PP, placenta previa; ART, assisted reproductive technology; Period, study period; Retro, retrospective study; Nationwide, nationwide study; Type, type of study; --, not applicable; Frozen, frozen embryo transfer; Fresh, fresh embryo transfer; Match, patient background matched study; No., number; END PAS, the study examined the effect of endometriosis on the prevalence of PAS; ART PP, the study determined the effect of ART on the prevalence of placenta previa; ART PAS, the study investigated the influence of ART on the prevalence of placenta accreta spectrum; ART_end; women with endometriosis conceived by ART; ART_non-end; women without endometriosis conceived by ART; SC_end; Spontaneous conception with endometriosis; SC_non-end; Spontaneous conception without endometriosis; HRC, hormone replacement cycle; NC, natural cycle; Unk, unknown; AUS, Australia; BEL, Belgium; CHE, Switzerland; CHN, China; DNK, Denmark; ESP, Spain; EST, Estonia; FIN, Finland; FRN, France; GBR, United Kingdom; IRN, Iran; ISR, Israel; ITA, Italia; JPN, Japan; NOR, Norway; POL, Poland; SVN, Slovenia; SWE, Sweden; USA, United States of America.

Supplemental Table S2. Metadata of studies (the rate of placenta previa: ART *versus* No ART and SC).

	Year	Exp	Control	ART End	No ART End	End ART	Non-End ART	Exp Previa	Control Previa	Adjusted OR (95%CI)	Exp End	Control End	Exp Previa	Control Previa	No. Exp	No. Control
Wang Y[1]	2021	ART	No ART	--	--	--	--	12376 (4.6%)	325504 (2%)	--	--	--	12376	325504	269738	16609990
Wang J[5]	2021	ART	No ART	74/1241 (6.0%)		74 (100%)		47 (3.8%)	94 (1.4%)	9.33 (4.22-20.62)	74	--	47	94	1241	6832
Epelboin S[6,a]	2021	ART_end	SC_end	100%	100%	6934/38035 (18.2%)	--	330 (4.8%)	520 (1.7%)	2.43 (2.10-2.82)	38035	--	330	520	6934	31101
Cochrane E[7]	2020	ART	SC	--	--	--	--	5 (4.2%)	1 (0.4%)	--	--	--	5	1	120	240
Salmanian B[9]	2020	ART	No ART	--	--	--	--	10 (1.8%)	271 (0.7%)	--	--	--	10	271	571	36890
Shinohara S[10]	2020	ART	SC	--	--	--	--	3 (0.7%)	18 (1%)	--	--	--	3	18	442	1764
Farella M[11,a]	2020	ART_end	SC_end	100%	100%	199/535 (37.2%)	--	7 (3.5%)	2 (0.6%)	--	535	0	7	2	199	336
Petersen SH[12]	2020	ART	SC	--	--	--	--	2560(1.7%)	22410 (0.3%)	3.87 (3.70-4.04)	--	--	2560	22410	146998	6683580
Tanaka H[14]	2020	ART	No ART	--	--	--	--	34 (4.2%)	96 (1.6%)	1.96 (1.24–3.06)	--	--	34	96	811	6141
Miura M[16]	2019	ART	No ART	--	--	23/80 (28.8%)	343/2689 (12.8%)	--	--	2.71 (1.70–4.31)	23	57	--	--	366	2403
Santi E[17]	2019	ART	No ART	--	--			9 (3.2%)	68 (0.6%)	--	--	--	9	68	281	11488
Szymusik I[20]	2019	ART	SC	33/336 (9.1%)	--	--	--	11 (3.3%)	2 (0.6%)	--	33	--	11	2	336	308
Yang P[21]	2019	ART	No ART	--	--	--	--	191 (11.5%)	254 (7.6%)	--	--	--	191	254	1663	3326
Nagata C[22]	2019	ART	SC	--	--	--	--	66 (2.1%)	489 (0.5%)	2.44 (1.87-3.20)[b]	--	--	66	489	3147	90506
Rahu K[23]	2019	ART	SC	--	--	--	--	22 (1.2%)	51 (0.2%)	--	--	--	22	51	1778	33555
Sabban H[26]	2017	ART	No ART	--	--	--	--	200 (3.5%)	18801 (0.6%)	3.14(2.71-3.64)	--	--	200	18801	5773	3309991
Li H[27]	2017	ART_end	No ART_end	100%	100%	24/98 (24.5%)	--	2 (8.3%)	0 (0%)	--	24	74	2	0	24	74
Qin J[29]	2017	ART	No ART	--	--	--	--	--	--	4.11 (2.12-7.96)	--	--	--	--	1260	2480
Zhu L[31]	2016	ART	SC	--	--	--	--	185 (7%)	179 (3.4%)	2.23 (1.79–2.78)	--	--	185	179	2641	5282
Ginström E[35]	2016	ART	SC	--	--	--	--	531 (1.7%)	3443 (0.3%)	6.38 (5.31-7.66)[a]	--	--	531	3443	30566	1196394
Valenzuela B[36]	2016	ART	No ART	--	--	--	--	3 (1.3%)	5 (0.7%)	--	--	--	3	5	223	725
Yang X[41]	2014	ART	SC	--	--	--	--	48 (4.2%)	1087 (1%)	2.18 (1.62–2.94)	--	--	48	1087	1139	111264
Räisänen S[43]	2013	ART	No ART	--	--	--	--	91 (1.6%)	571 (0.2%)	--	--	--	91	571	5647	285357
Hayashi M[47]	2012	ART	SC	--	--	--	--	--	--	2.2 (1.68-2.87)	--	--	--	--	4570	4264
Healy DL[50]	2010	ART	No ART	--	--	--	--	174 (2.6%)	273 (1.1%)	2.34 (1.87–2.92)	--	--	174	273	6730	24619
Verlaenen H[51]	1995	ART	No ART	--	--	--	--	4 (2.9%)	0 (0%)	--	--	--	4	0	140	140
Hill GA[52]	1990	ART	No ART	--	--	--	--	4 (6.6%)	2 (1.7%)	--	--	--	4	2	61	121

[a] limited the cases with blastcyst transfer; [b] calculated by the authors. Some values listed above might be slightly different from the original values, as estimated by the authors. Abbreviations: Exp, Experimental; End, endometriosis; Previa, placenta previa; ART, assisted reproductive technology; --, not applicable; No., number; Exp Previa (%), the rate of placenta previa in the experimental group; Control Previa (%), the rate of placenta previa in the control group; Exp Previa; the number of women with placenta previa in the experimental group; Control Previa; the number of women with placenta previa in the control group; No. Exp, the number of included pregnant women in the experimental group; No. Control, the number of included pregnant women in the control group; ART End (%); the rate of endometriosis in women who conceived using ART; End ART, the rate of ART pregnancy in women with endometriosis; ART_end; women with endometriosis conceived by ART; SC_end; Spontaneous conception with endometriosis; OR, odds ratio, CI, confidence interval.

Supplemental Table S3. Metadata of studies (the effect of ART on the rate of placenta previa: sensitivity analysis).

Author	Year	Exp	Control	Exp End (%)	Control End (%)	End ART (%)	Non-End ART (%)	Exp Previa (%)	Control Previa (%)	Exp No. End	Control No. End	No. Exp	No. Control	Adjusted OR (95%CI)
**Endometriosis *versus* Non-endometriosis (women with ART)**														
Wang J[5]	2021	ART_end	ART_non-end	74/1167 (6.0%)	--	100%	100%	8 (10.8%)	39 (3.3%)	74	1167	74	1167	--
Fujii T[30]	2016	ART_end	ART_non-end	92/604 (15.2%)	--	100%	100%	8 (8.7%)	4 (0.8%)	92	512	92	512	15.41 (4.40-61.7)
Benaglia L[32]	2016	ART_end	ART_non-end	--	--	100%	100%	14 (5.9%)	3 (1.3%)	239	239	239	239	--
Jacques M[34]	2016	ART_end	ART_non-end	160/2315 (6.9%)	--	100%	100%	3 (2.7%)	3 (2.7%)	113	113	113	113	--
Takemura Y[45]	2013	ART_end	ART_non-end	53/318 (16.7%)	--	100%	100%	9 (17%)	4 (1.5%)	53	265	53	265	--
Benaglia L[48]	2012	ART_end	ART_non-end	--	--	100%	100%	3 (3.8%)	1 (0.6%)	78	156	78	156	--
Healy DL[50]	2010	ART_end	ART_non-end	1265/6730 (18.8%)	--	100%	100%	48 (3.8%)	126 (2.3%)	--	--	1265	5465	1.65 (1.18–2.32)
**ART *versus* SC (women with endometriosis)**														
Epelboin S[6]	2021	ART_end	SC_end	6934/38035(18.2%)	--	100%	0%	330 (4.8%)	520 (1.7%)	6934	31101	6934	31101	--
		ART_end	SC_non-end	--	--	100%	0%	330 (4.8%)	23002 (0.6%)	6934	0	6934	4083732	--
**Frozen ET *versus* Fresh ET**														
Zargar M[2]	2021	Frozen	Fresh	--	--	--	--	17 (4.8%)	16 (5.1%)	--	--	356	313	--
Shinohara S[10]	2020	Frozen	Fresh	--	--	--	--	2 (0.6%)	1 (0.9%)	--	--	330	112	--
Sakai Y[18]	2019	Frozen	Fresh[b]	--	--	--	--	9 (15%)	3 (11.1%)	--	--	60	27[b]	--
Ginström E[35]	2016	Frozen	Fresh	--	--	--	--	80 (1%)	451 (2%)	--	--	7795	22771	--
Takeshima K[37]	2016	Frozen	Fresh	--	--	--	--	656 (0.7%)	460 (0.9%)	--	--	90783	50445	--
Kaser DJ[38]	2015	Frozen	Fresh	--	--	--	--	7 (16.6%)	31 (19.8%)	--	--	42	157	--
Rombauts L[39]	2014	Frozen	Fresh	99/1586 (6.2%)	217 (7.4%)	--	--	33 (2.1%)	112 (3.8%)	99	217	1586	2951	--
Korosec S[40]	2014	Frozen	Fresh	43/211 (20.4%)	187 (20.4%)	--	--	0 (0%)	32 (3.5%)	43	187	211	915	--
Liu SY[44]	2013	Frozen[a]	Fresh[a]	--	--	--	--	7 (0.5%)	6 (0.3%)	--	--	1531[a]	1914[a]	
Healy DL[50]	2010	Frozen	Fresh	--	--	--	--	51 (2.5%)	123 (3.0%)	--	--	2045	4058	0.73 (0.51–1.04)[c]
**HRC *versus* NC (Frozen ET)**														
Hu KL[3]	2021	HRC	NC	122 (4.8%)	198 (5.2%)	--	--	14 (0.5%)	15 (0.4%)	122	198	2561	3790	0.58 (0.27–1.24)[c]
Asserhøj LL[4]	2021	HRC	NC	16 (5.0%)	15 (2.9%)	--	--	7 (2/0%)	15 (1.9%)	16	15	357	731	1.10 (0.46–2.62)
Saito K[19]	2019	HRC	NC	--	--	--	--	119 (0.5%)	66 (0.6%)	--	--	24225	10755	1.00 (0.64–1.56)
Rombauts L[39]	2014	HRC	NC	--	--	--	--	12 (3.4%)	21 (1.7%)	--		355	1231	--

[a] day 3 embryo; [b] two cases were FET; [c] calculated by the author. Some values listed above might be slightly different from the original values, as estimated by the authors. Abbreviations: Exp, Experimental; End, endometriosis; Exp End, the rate of endometriosis in the experimental group; Control End, the rate of endometriosis in the control group; Exp No. End, the number of women with endometriosis in the experimental group; Control No. End, the number of women with endometriosis in the control group; No. Exp, the number of included pregnant women in the experimental group; No. Control, the number of included pregnant women in the control group; Previa, placenta previa; ART, assisted reproductive technology; --, not applicable; ET, embryo transfer; No., number; OR, odds rate; CI, confidence interval; END PAS, the study examined the effect of endometriosis on the prevalence of PAS; ART PP, the study determined the effect of ART on the prevalence of placenta previa; ART PAS, the study investigated the influence of ART on the prevalence of placenta accreta spectrum; ART_end; women with endometriosis conceived by ART; ART_non-end; women without endometriosis conceived by ART; SC_end; Spontaneous conception with endometriosis; SC_non-end; Spontaneous conception without endometriosis; HRC, hormone replacement cycle; NC, natural cycle.

Supplemental Table S4. Metadata of studies (the effect of ART on the rate of placenta accreta spectrum disorder).

Author	Year	Exp	Control	Total	Exp End	Control End	Exp PASD	Control PASD	No. Exp	No. Control	Adjusted OR (95%CI)
**ART *versus* No ART and SC**											
Wang Y[1]	2021	ART	No ART	16879728	--	--	12655 (4.7%)	325816 (2.0%)	269738	16609990	--
Wang J[5]	2021	ART	No ART	8773	74	--	44 (3.5%)	148 (2.2%)	1241	6832	1.67 (1.16-2.41)[a]
Cochrane E[7]	2020	ART	SC	360	--	--	3 (2.5%)	0 (0%)	120	240	--
Modest AM[8]	2020	ART	No ART	28344	--	--	31 (2.2%)	81 (0.3%)	1418	26926	--
Salmanian B[9]	2020	ART	No ART	37461	--	--	12 (2.1%)	218 (0.6%)	571	36890	--
Tanaka H[14]	2020	ART	No ART	6952	--	--	18 (2.2%)	18 (0.3%)	811	6141	7.35 (3.20-16.6)
Kyozuka H[15]	2019	ART	No ART	90554	--	--	39 (1.5%)	163 (0.2%)	2658	87896	6.78 (4.54-10.14)
Zhu L[31]	2016	ART	SC	7923	--	--	197 (7.5%)	173 (3.3%)	2641	5282	2.37 (1.90–2.95)
Hayashi M[47]	2012	ART	SC	25069	--	--	37 (0.8%)[a]	13 (0.3%)[a]	4570	4264	--
Esh-Broder E[49]	2011	ART	SC	25193	--	--	12 (1.6%)	30 (0.1%)	752	24441	--
**Frozen ET *versus* Fresh ET**											
Sakai Y[18]	2019	Frozen	Fresh	87	--	--	19 (31.7%)	2 (7.4%)	60	27[b]	--
Takeshima K[37]	2016	Frozen	Fresh	141,228	--	--	321 (0.4%)	35 (0.1%)	90783	50445	--
Kaser DJ[38]	2015	Frozen	Fresh	199	--	--	16 (38.1%)	34 (21.7%)	42	157	3.20 (1.14-9.02)
Korosec S[40]	2014	Frozen	Fresh	1126	43	187	7 (3.3%)	29 (3.2%)	211	915	--
**HRC *versus* NC (Frozen ET)**											
Saito K[19]	2019	HRC	NC	34980	--	--	142 (0.6%)	11 (0.1%)	24225	10755	6.91 (2.87-16.66)

[a] calculated by the author [b] two cases were frozen. Some values listed above might be slightly different from the original values, as estimated by the authors. Abbreviations: Exp, Experimental; End, endometriosis; Exp End, number of women with endometriosis in experimental group; Control End, number of women with endometriosis in control group; Exp End, the rate of endometriosis in the experimental group; Control End, the rate of endometriosis in the control group; Exp PASD, the number of women with placenta accreta spectrum disorder in the experimental group; Control PASD, the number of women with placenta accreta spectrum disorder in the control group; No. Exp, the number of included pregnant women in the experimental group; No. Control, the number of included pregnant women in the control group; PASD, placenta accreta spectrum disorder; ART, assisted reproductive technology; SC, spontaneous conception; --, not applicable; ET, embryo transfer; No., number; HRC, hormone replacement cycle; NC, natural cycle; OR, odds ratio; CI, confidence interval.

Supplemental Table S5. Risk of bias assessment for the comparator study.

Authors	Confounding	Selection	Classification of intervention	Deviations from interventions	Missing data	Measurement of outcomes	Reported results	Overall bias
Wang Y[1]	Orange	Purple	Purple	Purple	Purple	Purple	Orange	Purple
Zargar M[2]	Red	Purple	Purple	Black	Black	Red	Purple	Red
Hu KL[3]	Orange	Purple	Purple	Black	Purple	Purple	Orange	Purple
Asserhøj LL[4]	Purple	Purple	Purple	Black	Black	Purple	Orange	Purple
Wang J[5]	Purple	Purple	Red	Black	Black	Purple	Purple	Purple
Epelboin S[6]	Purple	Purple	Red	Black	Black	Purple	Orange	Purple
Cochrane E[7]	Red	Red	Purple	Black	Black	Red	Red	Red
Modest AM[8]	Purple	Orange	Purple	Orange	Black	Purple	Purple	Purple
Salmanian B[9]	Purple	Orange	Purple	Orange	Black	Purple	Purple	Purple
Shinohara S[10]	Purple	Orange	Purple	Purple	Purple	Purple	Purple	Purple
Farella M[11]	Purple	Purple	Orange	Purple	Purple	Purple	Purple	Purple
Petersen SH[12]	Purple	Purple	Purple	Orange	Orange	Purple	Orange	Purple
Tanaka H[14]	Purple	Red	Red	Purple	Black	Red	Red	Red
Kyozuka H[15]	Purple	Orange	Red	Purple	Purple	Purple	Purple	Purple
Miura M[16]	Red	Purple	Purple	Black	Black	Purple	Purple	Purple
Santi E[17]	Purple	Purple	Purple	Purple	Black	Purple	Purple	Purple
Sakai Y[18]	Purple	Red	Orange	Purple	Black	Red	Red	Red
Saito K[19]	Orange	Purple	Orange	Orange	Orange	Purple	Purple	Purple
Szymusik I[20]	Red	Red	Red	Black	Black	Purple	Red	Red
Yang P[21]	Purple	Purple	Red	Black	Black	Purple	Purple	Red
Nagata C[22]	Purple	Orange	Red	Purple	Orange	Red	Purple	Purple
Rahu K[23]	Purple	Purple	Purple	Black	Red	Purple	Purple	Purple
Shmueli A[24]	Purple	Purple	Purple	Black	Black	Purple	Purple	Purple
Nirgianakis K[25]	Purple	Red	Orange	Black	Black	Purple	Orange	Purple
Sabban H[26]	Purple	Purple	Purple	Black	Black	Purple	Purple	Purple
Li H[27]	Red	Red	Orange	Black	Black	Red	Purple	Red
Berlac JF[28]	Purple	Purple	Red	Black	Black	Red	Orange	Purple
Qin J[29]	Purple	Purple	Purple	Black	Black	Purple	Purple	Purple
Fujii T[30]	Purple	Purple	Orange	Black	Black	Purple	Purple	Purple
Zhu L[31]	Orange	Purple	Red	Purple	Black	Red	Purple	Purple
Benaglia L[32]	Purple	Red	Purple	Black	Black	Purple	Purple	Purple
Jacques M[34]	Red	Red	Purple	Black	Black	Purple	Purple	Red
Ginström E[35]	Orange	Orange	Purple	Black	Black	Purple	Purple	Purple
Valenzuela B[36]	Purple	Purple	Purple	Black	Black	Purple	Purple	Purple
Takeshima K[37]	Purple	Orange	Orange	Purple	Black	Red	Purple	Purple
Kaser DJ[38]	Orange	Purple	Orange	Orange	Black	Purple	Purple	Purple
Rombauts L[39]	Orange	Purple	Purple	Black	Black	Red	Purple	Purple
Korosec S[40]	Red	Purple	Purple	Black	Black	Purple	Red	Red
Yang X[41]	Purple	Purple	Purple	Black	Black	Purple	Purple	Purple
Räisänen S[43]	Orange	Orange	Purple	Black	Orange	Purple	Purple	Purple
Liu SY[44]	Purple	Purple	Purple	Black	Black	Purple	Purple	Purple
Takemura Y[45]	Red	Purple	Purple	Black	Black	Red	Red	Red

Hayashi M[47]	Moderate	Low	Low	Low	Low	Serious	Moderate	Moderate
Benaglia L[48]	Moderate	Serious	Moderate	No information	No information	Moderate	Moderate	Moderate
Esh-Broder E[49]	Moderate	Moderate	Serious	Moderate	No information	Moderate	Moderate	Moderate
Healy DL[50]	Moderate	Moderate	Serious	No information	No information	Moderate	Moderate	Moderate
Verlaenen H[51]	Serious	Moderate	Serious	No information	No information	Moderate	Moderate	Serious
Hill GA[52]	Serious	Moderate	Serious	No information	No information	Moderate	Moderate	Serious

Updated and reproduced the data from Sci Rep. 2021;11:920 Matsuzaki S *et al.* Antenatal diagnosis of placenta accreta spectrum after in vitro fertilization-embryo transfer: a systematic review and meta-analysis / Supplemental Table S3. Risk of bias assessment for the comparator study.[53]

Risk of bias assessment was performed using the Risk Of Bias In Non-randomized Studies–of Interventions tool (ROBINS-I).[54-56]

- Low risk of bias (the study is comparable to a well-performed randomized trial with regard to this domain)
- Moderate risk of bias (the study is sound for a non-randomized study with regard to this domain but cannot be considered comparable to a well-performed randomized trial)
- Serious risk of bias (the study has some important problems in this domain)
- Critical risk of bias (the study is too problematic in this domain to provide any useful evidence on the effects of intervention.
- No information on how to base a judgment on the risk of bias for this domain.

References

1. Wang Y, Shi H, Chen L, et al. Absolute Risk of Adverse Obstetric Outcomes Among Twin Pregnancies After In Vitro Fertilization by Maternal Age. JAMA network open 2021;4:e2123634.
2. Zargar M, Dehdashti S, Najafian M, Choghakabodi PM. Pregnancy outcomes following in vitro fertilization using fresh or frozen embryo transfer. JBRA assisted reproduction 2021;25:570-74.
3. Hu KL, Zhang D, Li R. Endometrium preparation and perinatal outcomes in women undergoing single-blastocyst transfer in frozen cycles. Fertil Steril 2021;115:1487-94.
4. Asserhøj LL, Spangmose AL, Aaris Henningsen AK, et al. Adverse obstetric and perinatal outcomes in 1,136 singleton pregnancies conceived after programmed frozen embryo transfer (FET) compared with natural cycle FET. Fertil Steril 2021;115:947-56.
5. Wang J, Liu Q, Deng B, Chen F, Liu X, Cheng J. Pregnancy outcomes of Chinese women undergoing IVF with embryonic cryopreservation as compared to natural conception. BMC Pregnancy Childbirth 2021;21:39.
6. Epelboin S, Labrosse J, Fauque P, et al. Endometriosis and assisted reproductive techniques independently related to mother-child morbidities: a French longitudinal national study. Reprod Biomed Online 2020.
7. Cochrane E, Pando C, Kirschen GW, Soucier D, Fuchs A, Garry DJ. Assisted reproductive technologies (ART) and placental abnormalities. Journal of perinatal medicine 2020;48:825-28.
8. Modest AM, Toth TL, Johnson KM, Shainker SA. Placenta Accreta Spectrum: In Vitro Fertilization and Non-In Vitro Fertilization and Placenta Accreta Spectrum in a Massachusetts Cohort. Am J Perinatol 2020.
9. Salmanian B, Fox KA, Arian SE, et al. In vitro fertilization as an independent risk factor for placenta accreta spectrum. Am J Obstet Gynecol 2020;223:568.e1-68.e5.
10. Shinohara S, Hirata S, Suzuki K. Association between infertility treatment and intrauterine growth: a multilevel analysis in a retrospective cohort study. BMJ open 2020;10:e033675.
11. Farella M, Chanavaz-Lacheray I, Verspick E, et al. Pregnancy outcomes in women with history of surgery for endometriosis. Fertil Steril 2020;113:996-1004.
12. Petersen SH, Bergh C, Gissler M, et al. Time trends in placenta-mediated pregnancy complications after assisted reproductive technology in the Nordic countries. Am J Obstet Gynecol 2020;223:226.e1-26.e19.
13. Spangmose AL, Ginström Ernstad E, Malchau S, et al. Obstetric and perinatal risks in 4601 singletons and 884 twins conceived after fresh blastocyst transfers: a Nordic study from the CoNARTaS group. Hum Reprod 2020;35:805-15.
14. Tanaka H, Tanaka K, Osato K, et al. Evaluation of Maternal and Neonatal Outcomes of Assisted Reproduction Technology: A Retrospective Cohort Study. Medicina (Kaunas, Lithuania) 2020;56.
15. Kyozuka H, Yamaguchi A, Suzuki D, et al. Risk factors for placenta accreta spectrum: findings from the Japan environment and Children's study. BMC Pregnancy Childbirth 2019;19:447.
16. Miura M, Ushida T, Imai K, et al. Adverse effects of endometriosis on pregnancy: a case-control study. BMC Pregnancy Childbirth 2019;19:373.
17. Santi E, Nencini G, Cerni A, et al. The PLART study: incidence of preterm labor and adverse pregnancy outcomes after assisted reproductive techniques-a retrospective cohort study. Arch Gynecol Obstet 2019;300:911-16.
18. Sakai Y, Ono M, Iizuka T, et al. Embryo transfer associated with hormone replacement therapy cycles using assisted reproductive technology increases placenta accreta spectrum. The journal of obstetrics and gynaecology research 2019;45:2394-99.
19. Saito K, Kuwahara A, Ishikawa T, et al. Endometrial preparation methods for frozen-thawed embryo transfer are associated with altered risks of hypertensive disorders of pregnancy, placenta accreta, and gestational diabetes mellitus. Hum Reprod 2019;34:1567-75.
20. Szymusik I, Kosinska-Kaczynska K, Krowicka M, Sep M, Marianowski P, Wielgos M. Perinatal outcome of in vitro fertilization singletons - 10 years' experience of one center. Archives of medical science : AMS 2019;15:666-72.
21. Yang P, Kang H, Ma C, Wei Y, Tao L, Wu Z. Risk of preterm delivery in singletons conceived by in vitro fertilization. Gynecological endocrinology : the official journal of the International Society of Gynecological Endocrinology 2019;35:661-64.
22. Nagata C, Yang L, Yamamoto-Hanada K, et al. Complications and adverse outcomes in pregnancy and childbirth among women who conceived by assisted reproductive technologies: a nationwide birth cohort study of Japan environment and children's study. BMC Pregnancy Childbirth 2019;19:77.
23. Rahu K, Allvee K, Karro H, Rahu M. Singleton pregnancies after in vitro fertilization in Estonia: a register-based study of complications and adverse outcomes in relation to the maternal socio-demographic background. BMC Pregnancy Childbirth 2019;19:51.
24. Shmueli A, Salman L, Hiersch L, et al. Obstetrical and neonatal outcomes of pregnancies complicated by endometriosis. J Matern Fetal Neonatal Med 2019;32:845-50.
25. Nirgianakis K, Gasparri ML, Radan AP, et al. Obstetric complications after laparoscopic excision of

posterior deep infiltrating endometriosis: a case-control study. Fertil Steril 2018;110:459-66.
26. Sabban H, Zakhari A, Patenaude V, Tulandi T, Abenhaim HA. Obstetrical and perinatal morbidity and mortality among in-vitro fertilization pregnancies: a population-based study. Arch Gynecol Obstet 2017;296:107-13.
27. Li H, Zhu HL, Chang XH, et al. Effects of Previous Laparoscopic Surgical Diagnosis of Endometriosis on Pregnancy Outcomes. Chinese medical journal 2017;130:428-33.
28. Berlac JF, Hartwell D, Skovlund CW, Langhoff-Roos J, Lidegaard O. Endometriosis increases the risk of obstetrical and neonatal complications. Acta Obstet Gynecol Scand 2017;96:751-60.
29. Qin J, Sheng X, Wu D, et al. Adverse Obstetric Outcomes Associated With In Vitro Fertilization in Singleton Pregnancies. Reproductive sciences (Thousand Oaks, Calif) 2017;24:595-608.
30. Fujii T, Wada-Hiraike O, Nagamatsu T, et al. Assisted reproductive technology pregnancy complications are significantly associated with endometriosis severity before conception: a retrospective cohort study. Reproductive biology and endocrinology : RB&E 2016;14:73.
31. Zhu L, Zhang Y, Liu Y, et al. Maternal and Live-birth Outcomes of Pregnancies following Assisted Reproductive Technology: A Retrospective Cohort Study. Scientific reports 2016;6:35141.
32. Benaglia L, Candotti G, Papaleo E, et al. Pregnancy outcome in women with endometriosis achieving pregnancy with IVF. Hum Reprod 2016;31:2730-36.
33. Wennberg AL, Opdahl S, Bergh C, et al. Effect of maternal age on maternal and neonatal outcomes after assisted reproductive technology. Fertil Steril 2016;106:1142-49.e14.
34. Jacques M, Freour T, Barriere P, Ploteau S. Adverse pregnancy and neo-natal outcomes after assisted reproductive treatment in patients with pelvic endometriosis: a case-control study. Reprod Biomed Online 2016;32:626-34.
35. Ginström Ernstad E, Bergh C, Khatibi A, et al. Neonatal and maternal outcome after blastocyst transfer: a population-based registry study. Am J Obstet Gynecol 2016;214:378.e1-78.e10.
36. Valenzuela-Alcaraz B, Crispi F, Manau D, et al. Differential effect of mode of conception and infertility treatment on fetal growth and prematurity. J Matern Fetal Neonatal Med 2016;29:3879-84.
37. Takeshima K, Jwa SC, Saito H, et al. Impact of single embryo transfer policy on perinatal outcomes in fresh and frozen cycles-analysis of the Japanese Assisted Reproduction Technology registry between 2007 and 2012. Fertil Steril 2016;105:337-46.e3.
38. Kaser DJ, Melamed A, Bormann CL, et al. Cryopreserved embryo transfer is an independent risk factor for placenta accreta. Fertil Steril 2015;103:1176-84.e2.
39. Rombauts L, Motteram C, Berkowitz E, Fernando S. Risk of placenta praevia is linked to endometrial thickness in a retrospective cohort study of 4537 singleton assisted reproduction technology births. Hum Reprod 2014;29:2787-93.
40. Korosec S, Ban Frangez H, Verdenik I, et al. Singleton pregnancy outcomes after in vitro fertilization with fresh or frozen-thawed embryo transfer and incidence of placenta praevia. BioMed research international 2014;2014:431797.
41. Yang X, Li Y, Li C, Zhang W. Current overview of pregnancy complications and live-birth outcome of assisted reproductive technology in mainland China. Fertil Steril 2014;101:385-91.
42. Ishihara O, Araki R, Kuwahara A, Itakura A, Saito H, Adamson GD. Impact of frozen-thawed single-blastocyst transfer on maternal and neonatal outcome: an analysis of 277,042 single-embryo transfer cycles from 2008 to 2010 in Japan. Fertil Steril 2014;101:128-33.
43. Räisänen S, Randell K, Nielsen HS, et al. Socioeconomic status affects the prevalence, but not the perinatal outcomes, of in vitro fertilization pregnancies. Hum Reprod 2013;28:3118-25.
44. Liu SY, Teng B, Fu J, Li X, Zheng Y, Sun XX. Obstetric and neonatal outcomes after transfer of vitrified early cleavage embryos. Hum Reprod 2013;28:2093-100.
45. Takemura Y, Osuga Y, Fujimoto A, et al. Increased risk of placenta previa is associated with endometriosis and tubal factor infertility in assisted reproductive technology pregnancy. Gynecological endocrinology : the official journal of the International Society of Gynecological Endocrinology 2013;29:113-5.
46. Sazonova A, Kallen K, Thurin-Kjellberg A, Wennerholm UB, Bergh C. Obstetric outcome in singletons after in vitro fertilization with cryopreserved/thawed embryos. Hum Reprod 2012;27:1343-50.
47. Hayashi M, Nakai A, Satoh S, Matsuda Y. Adverse obstetric and perinatal outcomes of singleton pregnancies may be related to maternal factors associated with infertility rather than the type of assisted reproductive technology procedure used. Fertil Steril 2012;98:922-8.
48. Benaglia L, Bermejo A, Somigliana E, et al. Pregnancy outcome in women with endometriomas achieving pregnancy through IVF. Hum Reprod 2012;27:1663-7.
49. Esh-Broder E, Ariel I, Abas-Bashir N, Bdolah Y, Celnikier DH. Placenta accreta is associated with IVF pregnancies: a retrospective chart review. Bjog 2011;118:1084-9.
50. Healy DL, Breheny S, Halliday J, et al. Prevalence and risk factors for obstetric haemorrhage in 6730 singleton births after assisted reproductive technology in Victoria Australia. Hum Reprod 2010;25:265-74.
51. Verlaenen H, Cammu H, Derde MP, Amy JJ. Singleton pregnancy after in vitro fertilization:

expectations and outcome. Obstetrics and gynecology 1995;86:906-10.
52. Hill GA, Bryan S, Herbert CM, 3rd, Shah DM, Wentz AC. Complications of pregnancy in infertile couples: routine treatment versus assisted reproduction. Obstetrics and gynecology 1990;75:790-4.
53. Matsuzaki S, Nagase Y, Takiuchi T, et al. Antenatal diagnosis of placenta accreta spectrum after in vitro fertilization-embryo transfer: a systematic review and meta-analysis. Scientific reports 2021;11:9205.
54. Sterne JA, Hernan MA, Reeves BC, et al. ROBINS-I: a tool for assessing risk of bias in non-randomised studies of interventions. BMJ 2016;355:i4919.
55. Danna SM, Graham E, Burns RJ, Deschenes SS, Schmitz N. Association between Depressive Symptoms and Cognitive Function in Persons with Diabetes Mellitus: A Systematic Review. PLoS One 2016;11:e0160809.
56. ROBINS-I detailed guidance (2016). https://www.riskofbias.info/welcome/home/current-version-of-robins-i/robins-i-detailed-guidance-2016. (accessed 11/20/2021).
