# Supplementary material for: Placenta Accreta Spectrum Disorder Complicated with Endometriosis: Systematic Review and Meta-Analysis
Source: Biomedicines. 2022 Feb 6;10(2):390. doi: 10.3390/biomedicines10020390 (PMC8962380; doi:10.3390/biomedicines10020390)
Supplement: Supplementary file 1 [file biomedicines-10-00390-s001.zip › Supplemental Figure S1.pdf]

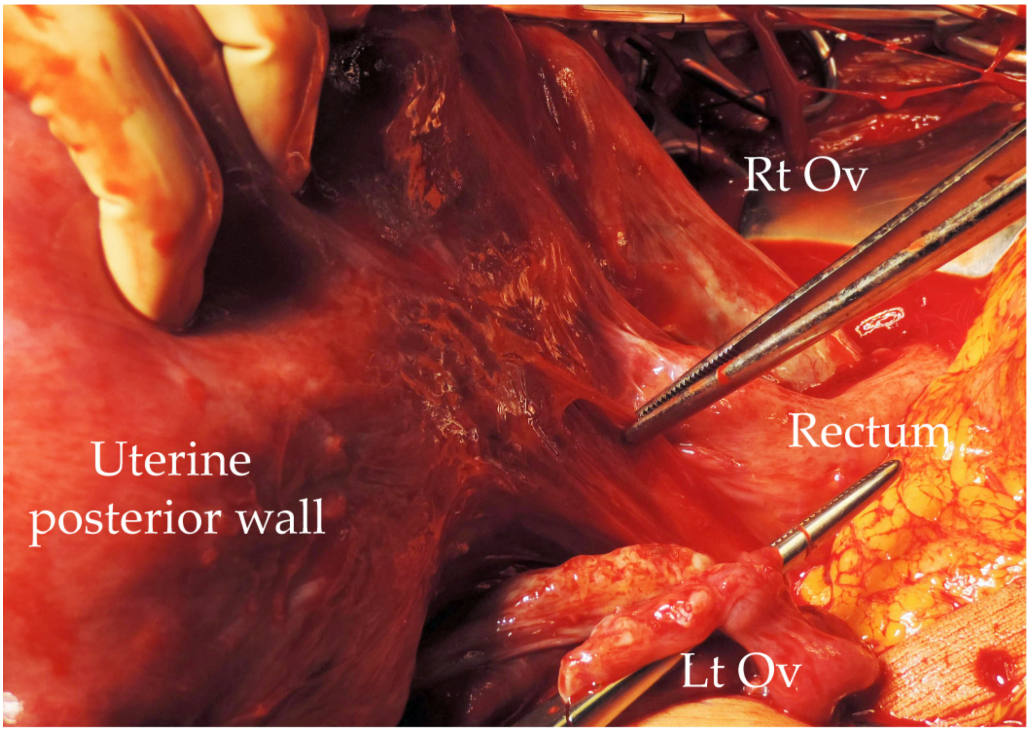

Supplemental Figure S1. Intraoperative image of placenta accreta spectrum complicated with endometriosis during cesarean hysterectomy.

A strong adhesion between the uterine posterior wall and rectum is observed. Massive hemorrhage (approximately 4800 mL) caused by the severe adhesion is observed. Abbreviations: Rt, right; Lt, left; Ov, ovary. Modified from Matsuzaki S *et al.* Horizontal cervix as a novel sign for predicting adhesions on the posterior extrauterine wall in cases of placenta previa. J Clin Med. 2019 Dec 4;8:2141.
